# Liver and bladder morbidity in a *Schistosoma mansoni* and *haematobium* co-endemic area in the Democratic Republic of Congo

**DOI:** 10.1371/journal.pntd.0013999

**Published:** 2026-02-17

**Authors:** Sylvie Linsuke, Joule Madinga, Clémentine Roucher, Mamy-Irène Miantezila, Sylvain Baloji, Michel Disonama, Jean-Pierre Van Geertruyden, Katja Polman, Pascal Lutumba

**Affiliations:** 1 Department of Epidemiology, National Institute of Biomedical Research (INRB), Kinshasa, the Democratic Republic of the Congo; 2 Global Health Institute, Faculty of Medicine, University of Antwerp, Anvers, Belgium; 3 Faculty of Medicine, University of Kikwit, Kikwit, the Democratic Republic of the Congo; 4 Department of Public Health, Institute of Tropical Medicine, Antwerp, Belgium; 5 Department of Internal Medicine, University of Kinshasa, Kinshasa, the Democratic Republic of the Congo; 6 National Control Program against sleeping sickness, Kinshasa, the Democratic Republic of the Congo; 7 Ministry of Health, rural health zone of Kwilu-Ngongo, Kwilu-Ngongo, the Democratic Republic of the Congo; 8 Department of Health Sciences, VU University Amsterdam, Amsterdam, The Netherlands; 9 Department of Tropical Medicine, University of Kinshasa, Kinshasa, the Democratic Republic of the Congo; International Atomic Energy Agency, AUSTRIA

## Abstract

**Background:**

Both *Schistosoma* (*S.) mansoni* and *S. haematobium* are co-endemic in many regions of the Democratic Republic of the Congo (DRC). However, little is known about the clinical implications of mixed *Schistosoma* infections for the affected communities. This study determined *Schistosoma*-related morbidity patterns in single and mixed *Schistosoma* infections in DRC.

**Methods:**

Between November 2015 and March 2016, we conducted a community-wide study in the rural community of Kifwa II village, west of the DRC. According to WHO guidelines, *Schistosoma-specific* morbidity was assessed using ultrasound. Data were summarized using descriptive statistics, the Chi-square test, and logistic regression.

**Principal findings:**

Ultrasound examinations were performed on 825 individuals aged 1–80 years. The results indicated that 68% (559) of the participants had bladder morbidity, with a higher prevalence observed in those with mixed infections compared to those with single *S. haematobium* infection (p = 0.001). The prevalence of liver morbidity was 64.6% (533), showing no difference between those with mixed and single *S. mansoni* infections (p = 0.174). Bladder morbidity was significantly associated with age, reflected by increasing odds ratios for different age groups: 1.6 (p = 0.035) for those aged 11–20 years, 1.7 (p = 0.046) for those aged 21–30 years, 2.2 (p = 0.005) for those aged 41–50 years, 3.2 (p = 0.001) for those aged 51–60 years, and 3.6 (p = 0.006) for those aged over 60 years. Similarly, liver morbidity was significantly associated with age, showing increasing odds ratios for different age groups: 1.7 (p = 0.026) for 21–30 years, 3.2 (p < 0.001) for 41–50 years, 4.5 (p < 0.001) for 51–60 years, and 3.1 (p = 0.011) for those over 60 years. However, mixed infection was associated with a significantly increased risk of bladder morbidity (aOR=2.3; p < 0.001), but not liver morbidity (aOR=1.4; p = 0.125).

**Conclusion:**

*Schistosoma*-related morbidity is common in the study area, impacting individuals of all ages and calling for regular mass drug administration, considering the adult population, improved WASH infrastructure, and health education to encourage behavioural change. Additionally, mixed infections have a positive effect on bladder morbidity, with possible implications for disease control.

## Author summary

Schistosomiasis is a chronic and debilitating parasitic disease that affects 254 million people worldwide. Chronic infection with *S. mansoni* and *S. haematobium* can lead to organ damage of the liver and the urinary tract, respectively. Both species are endemic to the Democratic Republic of the Congo (DRC) and coexist in several regions, leading to the occurrence of mixed infections. However, little is known about the magnitude of liver and bladder schistosomiasis-related morbidity in these co-endemic areas and how this is affected by mixed infections. We investigated specific morbidity related to *S. mansoni* and *S. haematobium* in a co-endemic region and compared the risk of morbidity between people harboring mixed *Schistosoma* infections and those with single infections. For this, we screened 825 inhabitants of Kifwa II village in western DRC for *Schistosoma* infections and related specific organ morbidity. This study showed that liver and bladder morbidity were highly prevalent in this community, and affected all age groups. Also, bladder morbidity was more common in mixed infections than in single *S. haematobium* infections while mixed infections did not affect liver morbidity. The interaction effect between *S. haematobium* and *S. mansoni* on bladder morbidity could have implications for schistosomiasis control in the regions where both species coexist.

## Background

Schistosomiasis is a chronic and devastating disease responsible for substantial morbidity, especially in rural areas of Sub-Saharan Africa (SSA), where the disease remains a major public health problem [1]. Globally, an estimated 779 million persons worldwide are at risk of schistosomiasis, 254 million are infected and 20 million have severe consequences [2]. In 2021, the global burden of schistosomiasis was estimated at 1,862,252 (1,038,122-2,984,204) disability-adjusted life-years lost; 90% of this burden occurred in SSA [3,4]. Two major *Schistosoma* species prevail in SSA: *Schistosoma* (*S.) mansoni* and *S. haematobium*, responsible for intestinal and urogenital schistosomiasis, respectively [1].

In endemic areas, chronic infection with schistosomiasis can cause important health problems, mostly among children, including anemia, malnutrition, stunting, and impaired cognitive development [5–8]. Moreover, if left untreated, the inflammatory and granulomatous immune responses of the host’s organism to worm eggs trapped in organ tissues can lead to permanent organ damage of the urinary or intestinal tracts [5,9–18]. Lesions are located according to the oviposition site of the *Schistosoma* species involved: *S. mansoni* causes liver lesions, whereas *S. haematobium* causes bladder lesions [1,9].

Schistosomiasis-related morbidity is heterogeneously distributed within the human population and is generally associated with high infection intensity [19–21]. Bladder morbidity has been reported to be more frequent in children, while liver morbidity is more frequent in adults [22–25]. Regarding gender, males are reportedly more prone to schistosomiasis-related morbidity compared to females [22–26]. However, most of these epidemiological studies were conducted in mono-endemic areas or reported only one *Schistosoma* species [13,27–33], and little is known about schistosomiasis-related morbidity in areas co-endemic for both *S. mansoni* and *S. haematobium*, where mixed infections are commonplace. The available studies showed that overall mixed infection with *S. mansoni* and *S. haematobium* reduces the risk of liver and splenic pathology while increasing urogenital pathology [34,35].

The Democratic Republic of the Congo (DRC) is endemic to schistosomiasis, with 15 million infected persons (7.3% within SSA and 6.6% worldwide) [36–39]. Both *S. mansoni* and *S. haematobium* have been prevalent in the country, with a large overlap of *S. haematobium*- and *S. mansoni*-endemic areas [37,40]. However, there is a paucity of published information on the prevalence of *S. mansoni* and *S. haematobium* mixed infections in the country. In a recent study conducted in the same region as for the current study, we found 38.4% of mixed infections among school-aged children [40]. Similarly, little is known about the extent of *Schistosoma*-related morbidity in the country for both single and mixed infections. Data reporting morbidity due to *Schistosoma* infections in the DRC is old and limited to clinical observations [28,40–50], except for one study conducted in Ituri in the northeast of DRC on morbidity related to *S. mansoni* using ultrasound data [27]. So far, no studies have addressed the morbidity of *S. haematobium* or mixed infections in DRC. The current study aims to determine patterns of *S. haematobium*-specific bladder morbidity and *S. mansoni*-specific hepatic morbidity in a co-endemic rural community and compare morbidity in those with mixed *Schistosoma* infections to those with single infections.

## Materials and methods

### Ethical statement

Ethical approval was received from the Ethics Committee of the School of Public Health of Kinshasa University (approval No ESP/CE/050/2014) in DRC, the Institutional Review Board of the Institute of Tropical Medicine of Antwerp (approval No 852/12) and the University of Antwerp (approval No 12/50/423) in Belgium. The study was also authorized by DRC’s Ministry of Health (MoH). Written informed consent was obtained from all participants before their inclusion. For participants under 18 years, written informed consent was obtained from their parents or legal guardians with the verbal consent of the children if aged more than 14 years.

### Study setting and design

**Fig 1:**
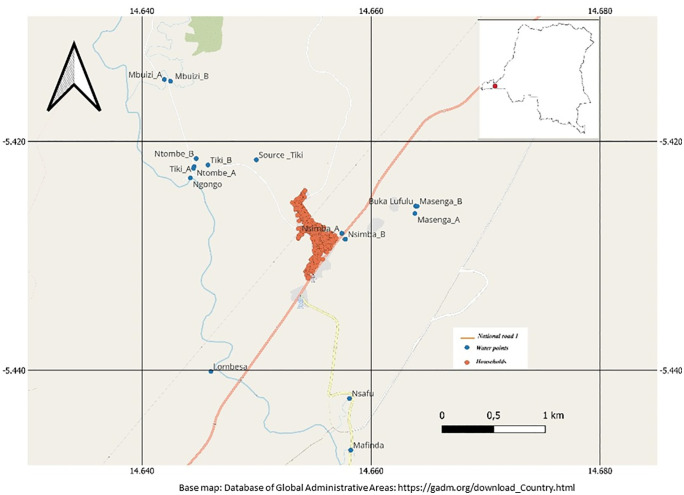
Map of the Study site showing Kifua II village in the DRC. (The base layer of the figure in this paper was obtained from the database of Global Administrative Areas: https://geodata.ucdavis.edu/gadm/gadm4.1/shp/gadm41_COD_shp.zip).

A community-based cross-sectional study was conducted between November 2015 and March 2016 among communities living in a rural area of Kifwa II village (Fig 1), located in the western part of the DRC between -5°25’1” degrees latitude south and 14°39’19.1” degrees longitude east. Its surface is 9965 km^2^ with an estimated population of around 1,400 inhabitants. The climate is tropical with two seasons. The dry season is between June and September, and the rainy season is between October and May, interrupted by a small dry season in February. The village is located alongside the Ngongo River and is home to several water points, including Ngongo, Mbwizi, Tiki, Ntombe, Masenga, Nsimba, Lombesa, N’Samfu, Buka Lufulu, and Kiminga (Fig 1). These various sources are also used for irrigation activities, as most residents are involved in agriculture, which remains their primary economic activity. Besides, the residents of this village do not have access to water in their households and instead rely on the various rivers (Fig 1) for their water supply. Based on information collected from local health zone leaders, Kifwa II village is endemic to both *S. mansoni and S. haematobium*. However, before the current study there were no public health interventions targeting schistosomiasis in the area.

### Data collection and processing

The study participants included community members of Kifua II village. Eligible participants were residents aged ≥ 1 year who had lived in the area for at least one year, had no recent history of taking anthelminthic drugs, and willing to provide urine and stool samples. Those who refused to give consent were not included in the study. Before starting the survey, the researchers’ team visited the study area and informed the chief of the village about the study objectives. The heads of the households were also informed about the study. Eligible participants who gave informed consent (or parental consent for minors) were invited to provide two stool and two urine samples on consecutive days within one week for the diagnosis of helminth infections (*Schistosoma spp*. and other intestinal helminths). Each participant received two pre-labeled, clean and dry 50ml containers with a unique identification number (ID) and was instructed to fill them with at least half volume of urine and stool respectively. Clear instructions for the hygienic collection procedure were given. Samples were collected in the morning between 8:00 AM and 12:00 PM, as most of the people left the village for farming activities thereafter. They were then logged on a registration form upon receipt for proper tracking, and transferred in cool boxes to the laboratory of IME/Kimpese for analyses according to the standard operating procedures of the study. After completing the parasitology assessment, all participants were invited to the village’s health center for an ultrasound examination to detect organ pathologies due to *S. mansoni* and *S. haematobium* infections. Demographic data were also recorded from all participants using a structured pre-tested questionnaire.

### Parasitology examination

Two stool and two urine samples were collected on two consecutive days and processed microscopically using standard parasitological procedures as recommended by the WHO guidelines for estimating infection prevalence and intensity [51].

For stool, duplicate Kato-Katz thin smears were prepared from each sample (2x2 smears per participant) and examined microscopically for egg counts expressed as eggs per gram (epg) [52]. Briefly, the stool was pressed through a metal mesh (Sterlitech Corporation, nylon screen, 100 mesh) to remove large particles. Then, a portion of 25 mg of the sieved fecal material was transferred to produce thick Kato-Katz smears on a slide. This was covered with a piece of cellophane soaked in a glycerol and methylene blue solution. All slides were examined under a microscope within 24 hours by two independent trained technicians to detect and count *S. mansoni* eggs. Infection load was calculated by finding the average of the number of egg counts from 4 slides, multiplied by 24 to estimate the epg of stool. Intensity of *S. mansoni* infection was reported as the number of eggs per grams (epg) and classify as per WHO guidelines [51] into light (1–99 epg), moderate (100–399 epg), or heavy (≥400 epg) infection.

For urine, 10 mL samples were processed using membrane filtration (2 × 1 slide per participant) and examined for *S. haematobium* eggs [53,54]. As previously described, 10 ml of urine from each sample was collected in a syringe after gently shaking and filtering through a 12 µm Isopore membrane filter. The membrane filter was then placed on a slide, dropped with Lugol’s iodine, and examined immediately under a microscope. The number of eggs observed in the preparation was recorded. Intensity of *S. haematobium* was reported as the number of eggs per 10 ml of urine (ep10 ml), and infection intensity was categorised per WHO guidelines [51] into light (1–49 eggs) or heavy (≥50 eggs).

To ensure quality, a senior technician who was blinded to the results of the other two technicians re-examined 10% of the randomly selected slides of all positive and negative stool and urine samples.

### Ultrasound examination

Ultrasound examinations were performed on all participants using a portable ultrasound machine (SonoAcerR3) with a convex 3.5 MHz transducer. All ultrasound examinations were performed independently by two trained physicians who were not informed about the parasitological results of the participants. Based on the Niamey protocol, *S. mansoni* and *haematobium*-related morbidity indicators were evaluated [55,56]. *S. mansoni*-specific liver morbidity was determined by the presence of the liver image pattern. Liver image pattern was graded to A, B, C, D, E, and F (A = no sign of periportal fibrosis, B = incipient periportal fibrosis not excluded, C = periportal fibrosis possible, D = periportal fibrosis probable, E = advanced periportal fibrosis, and F = very advanced periportal fibrosis). Based on the degree of fibrosis, liver image patterns A and B were considered normal or not specific to *S mansoni* infection, and liver image patterns C, D, E, and F were considered *S. mansoni*-specific morbidity. For *S. haematobium*-specific morbidity, urinary bladder morbidity was determined using the WHO score [55,56]. A score of 0 was considered normal ultrasound or absence of bladder anomalies, and a score ≥1 indicated bladder abnormalities, which increased in severity (scores 1, 2, and 3), and was considered as *S. haematobium*-specific urinary bladder morbidity.

### Data analysis

Data were double-entered into Access software, and statistical analyses were performed using Stata software 12.0. Descriptive analysis was applied to summarize the data. These include frequency and proportions for categorical variables, means with standard deviations, or medians with interquartile range for continuous variables, depending on the distribution. The geometric mean of egg counts (GM epg or GM ep 10ml for *S. mansoni* and *S. haematobium*, respectively) was calculated individually for participants who were positive for *Schistosoma* infections, after log-transformation to determine *Schistosoma* infections intensity. The chi-square test (χ2) was estimated to compare proportions between groups. Logistic regression using generalized linear models was used to assess the effect of participant age on the bladder and liver morbidity positivity rate. Besides, multivariable logistic regression models were developed to determine the predictors of schistosomiasis-related morbidity. Gender, age, *S. haematobium* infection, and *S. mansoni* infection were included as potential risk factors. To assess the impact of mixed infections on morbidity, participants were categorized into four groups: (i) single *S. haematobium* infection, (ii) single *S. mansoni* infection, (iii) co-infection with *S. haematobium* and *S. mansoni*, and (iv) uninfected individuals, who served as the reference group. Only variables with a likelihood ratio p-value <0.25 were included in the multivariable analysis for each model. The final model was selected using a backward elimination based on the smallest Akaike information criterion (AIC). The results were reported as adjusted OR (aOR), and a p-value of <0.05 is considered significant.

## Results

### General characteristics of participants

Complete data (parasitological and ultrasound data) were obtained from 825 participants, of which 393 (47.6%) were males and 432 (52.4%) were females. The median age of all participants was 15 years (IQR: 7–36 years; Range: 1–80 years) (Table 1). Parasitological examination revealed the overall prevalence rate of *Schistosoma* infection at 77.2% (637/825), with *S. haematobium* at 57.9% (478/825), *S. mansoni* at 57.6% (457/825 of whom 38.3% (316/825) were mixed infections. Distributions of single and mixed *Schistosoma* infections according to gender and age are summarized in Table 1.

**Table 1:** General characteristics of study participants and Schistosomiasis prevalence pattern according to gender and age.

Variables	All		*Schistosoma spp*		*S. haematobium*		*S. mansoni*		*Mixed infection*	
	n	% (95% CI)	n	% (95% CI)	n	% (95% CI)	n	% (95% CI)	n	% (95% CI)
Total of participants	**825**		**637**	**77.2 (74.3-80.1)**	**478**	**57.9 (54.6-61.3)**	**475**	**57.6 (54.2-61.0)**	**316**	**38.3 (34.9-41.6)**
Gender										
Female	432	52.4 (48.9-55.8)	339	53.2 (49.3-57.1)	260	54.4 (44.3-54.8)	245	51.6 (47.1-56.1)	166	52.5 (47.0-58.1)
Male	393	47.6 (44.2-51.1)	298	46.8 (42.8-50.7)	218	45.6 (41.1-50.1)	230	48.4 (43.9-52.9)	150	47.5 (41.9-52.9)
Median age, years (IQR)	15 (7-36)									
Age groups (years)										
0-10	321	38.9 (35.6-42.2)	238	37.4 (33.6-41.1)	192	40.2 (35.8-44.6)	157	33.1 (28.8-37.3)	111	35.1 (29.8-40.4)
11-20	151	18.3 (15.7-21.0)	136	21.4 (18.1-24.5)	116	24.3 (20.4-28.1)	127	26.7 (22.7-30.7)	107	33.9 (28.6-39.1)
21-30	93	11.3 (9.1-13.4)	79	12.4 (9.8-15.0)	61	12.8 (9.8-15.8)	52	10.9 (8.1-13.8)	34	10.8 (7.3-14.2)
31-40	90	10.9 (8.8-13.0)	65	10.2 (7.8-12.6)	42	8.8 (6.2-11.3)	50	10.5 (7.8-13.3)	27	8.5 (5.5-11.6)
41-50	88	10.7 (8.6-12.8)	67	10.5 (8.1-12.9)	38	7.9 (5.5-10.4)	54	11.4 (8.5-14.2)	25	7.9 (4.9-10.9)
51-60	50	6.1 (4.4-7.7)	32	5.0 (3.3-6.7)	16	3.4 (1.7-4.9)	22	4.6 (2.7-6.5)	6	1.9 (0.4-3.4)
>60	32	3.9 (2.6-5.2)	20	3.1 (1.8-4.5)	13	2.7 (1.3-4.2)	13	2.7 (1.3-4.2)	6	1.9 (0.4-3.4)

### *Schistosoma*-related specific morbidity prevalence and infection status

Overall, 67.8% (559/825) of the study participants had bladder morbidity, with the most important type of bladder morbidity score observed among the study participants being a score 2 (73.3%; 410/559), followed by the score ≥3 (25.8%; 144/559). A small proportion of participants, 0.9% (5/559), presented a bladder morbidity score of 1 (Table 2). Out of 559 individuals with bladder morbidity, 116 (20.8%) were not infected, 103 (18.4%) had a single *S. haematobium* infection, and 246 (44.0%) had a mixed infection (Table 2). Furthermore, the prevalence rate of bladder morbidity was higher among those with mixed infection compared to those with a single infection (χ2 = 11.1; p = 0.001).

**Table 2:** *S. haematobium*-associated bladder morbidity and Schistosoma species infection status among the study participants.

Morbidity	Urinary bladder			*S. haematobium* infection			*S. mansoni* infection			*Mixed infection*	
	Scores	n	%	n	%	GM EP/10ML (95% CI)	n	%	GM EPG (95% CI)	n	%
**Negative**	**0**	**266**	**32.2**	**129**	**27.0**	**7.2 (5.6-9.4)**	**135**	**28.4**	**78.7 (62.9-98.6)**	**70**	**22.2**
											
**Positive**	**≥ 1**	**559**	**67.8**	**349**	**73.0**	**12.0 (10.0-14.3)**	**340**	**71.6**	**103.7 (89.2-120.5)**	**246**	**77.8**
	1	5	0.9	2	0.6	23.5 (4.84e-06-1.14e + 08)	3	0.9	28.8 (1.2-697.4)	1	0.4
	2	410	73.3	232	66.5	10.3 (8.2-12.8)	243	71.5	91.9 (77.5-109)	162	65.9
	≥ 3	144	25.8	115	33.0	16.1 (12.0-21.5)	94	27.6	147.4 (108.2-200.8)	83	33.7
**Total**		**825**		**478**	**57.9**	**10.4 (8.9-12.1)**	**475**	**57.6**	**95.9 (84.6-108.7)**	**316**	**38.3**

The overall prevalence rate of liver morbidity among the study participants was 64.6% (533/825). Grade C (86.3%; 460/533) was the most common liver image pattern, followed by D (11.1%; 59/533) in the study participants. Severe liver morbidity (liver image pattern E and F) was only shown in 2.6% (14/533) of the study participants (Table 3). Of 533 participants who had liver morbidity, 113 (21.2%) were not infected, 114 (21.4%) had a single *S. mansoni* infection, and 207 (38.8%) had a mixed infection (Table 3). However, there was no difference in the prevalence rate of liver morbidity among those infected with a single *S. mansoni* and those with mixed infection (χ2 = 1.9; p = 0.174).

**Table 3:** *S. mansoni*-associated liver morbidity and Schistosoma species infection status among the study participants.

Morbidity	Liver Image Pattern			*S. mansoni infection*			*S. haematobium infection*			*Mixed infection*	
	Grades	n	%	n	%	GM EPG (95% CI)	n	%	GM EP/10ML (95% CI)	n	%
**Negative**	**A-B**	**292**	**35,4**	**154**	**32.4**	**99.3 (80.6-122.4)**	**172**	**36.0**	**12.0 (9.3-15.3)**	**109**	**34.5**
	A	237	81.2	127	82.5	109.2 (86.86-137.5)	143	83.1	12.4 (9.5-16.1)	93	85.3
	B	55	18.8	27	17.5	63.5 (38.6-104.5)	29	16.9	10.0 (4.9-20.3)	16	14.7
											
**Positive**	**C-F**	**533**	**64.6**	**321**	**67.6**	**94.2 (80.6-110.3)**	**306**	**64.0**	**9.7 (8.0-11.7)**	**207**	**65.5**
	C	460	86.3	283	88.2	91.3 (77.2-108.0)	274	89.5	10.3 (8.4-12.5)	189	91.3
	D	59	11.1	31	9.7	104.0 (61.6-175.4)	25	8.2	7.0 (3.3-14.4)	14	6.8
	E	7	1.3	3	0.9	370.9 (36.8-432.9)	5	1.6	3.5 (0.3-36.3)	2	1.0
	F	7	1.3	4	1.2	145.6 (39.8-533.1)	2	0.7	2.4 (0.2-32.2)	2	1.0
**Total**		**825**		**475**	**57.6**	**95.9 (84.6-108.7)**	**478**	**57.9**	**10.4 (8.9-12.1)**	**316**	**38.3**

Of the 825 individuals screened by ultrasound in this study, 690 presented with *Schistosoma*-related morbidity. Of these individuals, 149 (21.6%) tested negative for schistosomiasis, with no differences between this group and those who were infected. However, stratified analysis by gender and age showed no difference in infection prevalence by gender. Age, however, showed a significant difference in prevalence, particularly among those aged 11–20 (p = 0.001) and 51–60 (p = 0.029). Characteristics and distribution of individuals with any morbidity without infection are shown in Table 4.

**Table 4:** Distribution of participants with morbidity according to infection status, stratified by gender and age.

Variables	*Schistosoma*-related morbidity	*Schistosoma* infections status		
		Infected	Uninfected	P-value
Total of participants	**690**	**541 (78.4)**	**149 (21.6)**	0.065
Gender				
Female	360	289 (80.3)	71 (19.7)	0.212
Male	330	252 (76.4)	78 (23.6)	
Age group				
0-10	240	185 (77.1)	55 (22.9)	1
11-20	138	125 (90.6)	13 (9.4)	0.001
21-30	80	68 (85.0)	12 (15.0)	0.135
31-40	76	57 (75.0)	19 (25.0)	0.709
41-50	79	58 (73.4)	21 (26.6)	0.507
51-60	47	29 (61.7)	18 (38.3)	0.029
>60	30	19 (63.3)	11 (36.7)	0.103

### Age-related patterns of specific morbidity

**Fig 2:**
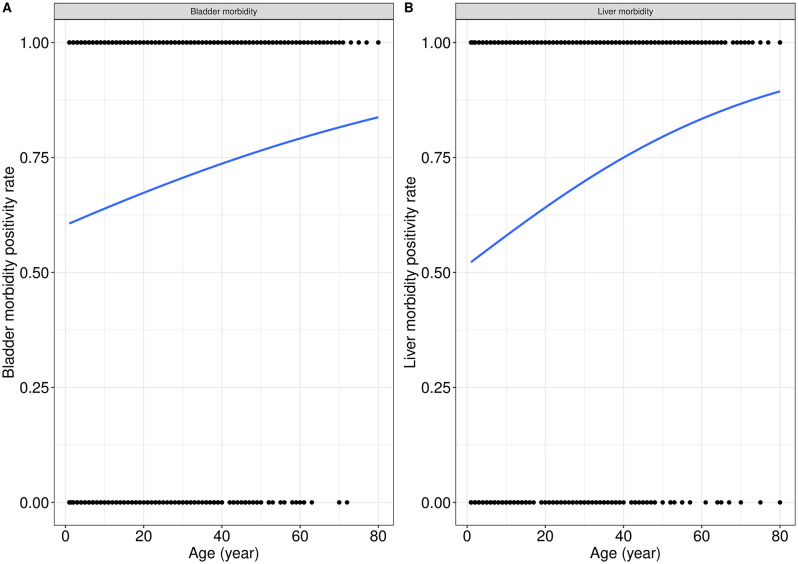
Association between age of participants and bladder and liver morbidity.

Fig 2A and 2B illustrates the effects of participant age on bladder and liver morbidity positivity rates. It can be observed from Fig 1A and 1B that bladder and liver morbidity positivity rates increased as participant age increased (X1=13.67, p=0.0002 (Fig. 2A), and X2= 38.3, p = 0.0001 (Fig. 2B)).

### Risk factors of bladder and liver morbidity

Bladder morbidity was significantly associated with age (Table 5), particularly in participants aged 11–20 years (aOR=1.6, p = 0.035), 21–30 years (aOR=1.7, p = 0.046), 41–50 years (aOR=2.2, p = 0.005), 51–60 years (aOR=3.2, p = 0.001), and over 60 years (aOR=3.6, p = 0.006). Liver morbidity was also strongly age-dependent, with significantly higher odds observed in participants aged 11–20 years (aOR=1.5, p = 0.048), 21–30 years (aOR=1.7, p = 0.026), 41–50 years (aOR=3.2, p < 0.001), 51–60 years (aOR=4.5, p < 0.001), and over 60 years (aOR=3.1, p = 0.011).

**Table 5:** Predictors of bladder and liver morbidity in Kifwa II community, DRC.

Variables	Bladder morbidity (n = 559)					Liver morbidity (n = 533)				
	n	cOR (95% CI)	P-value	aOR** (95% CI)	P-value	n	cOR (95% CI)	P-value	aOR** (95% CI)	P-value
**Gender**										
Female	286	Ref.				287	Ref.			
Male	273	1.2 (0.9-1.6)	0.317	1.3 (0.9-1.7)	0.120	246	0.8 (0.6-1.1)	0.250	0.9 (0.7-1.2)	0.553
**Age**										
0-10	190	Ref.				174	Ref.			
11-20	114	2.1 (1.4-3.3)	0.001	1.6 (1.0-2.3)	0.035	100	1.7 (1.1-2.5)	0.014	1.5 (1.0-2.3)	0.048
21-30	65	1.6 (1.0-2.6)	0.063	1.7 (1.0-2.8)	0.046	63	1.8 (1.1-2.9)	0.021	1.7 (1.0-2.8)	0.036
31-40	61	1.5 (0.9-2.4)	0.141	1.6 (1.0-2.7)	0.069	59	1.6 (1.0-2.6)	0.056	1.5 (1.0-2.6)	0.071
41-50	64	1.8 (1.1-3.1)	0.022	2.2 (1.3-3.7)	0.005	70	3.3 (1.9-5.8)	<0.001	3.1 (1.8-5.5)	<0.001
51-60	39	2.4 (1.2-4.9)	0.013	3.2 (1.6-6.6)	0.001	42	4.4 (2.0-9.7)	<0.001	4.4 (2.0-9.8)	<0.001
> 60	26	3.0 (1.2-7.5)	0.019	3.6 (1.4-9.2)	0.006	25	3.0 (1.3-7.2)	0.012	3.1 (1.3-7.4)	0.011
**Infection status**										
*No S. haematobum*	210	Ref.				227	Ref.			
*S. haematobium mono-infection*	349	1.8 (1.3-2.4)	<0.001	1.2 (0.8-1.9)	0.451	306	0.9 (0.7-1.3)	0.678	1.1 (0.7-1.8)	0.594
*No S. mansoni*	219	Ref.				212	Ref.			
*S. mansoni mono-infection*	340	1.5 (1.1-2.0)	0.006	0.8 (0.5-1.3)	0.374	321	1.4 (1.0-1.8)	0.038	1.5 (1.0-2.5)	0.070
*S.haematobium x S.mansoni u-interaction*				2.4 (1.3-4.4)	0.006**				0.8 (0.4-1.5)	0.448

*Signiﬁcant at p ≤ 0.05, cOR=crude odds ratio, aOR= adjusted odds ratio.

** adjusted for age & gender, stratified by mono and mixed Schistosoma infection due to interactions.

*** *Classic model with S. mansoni and S. haematobium infection status, adjusted for age.*

In unadjusted analysis, *S. haematobium* infection was significantly associated with bladder morbidity (cOR=1.8; 95% CI: [1.3–2.4]; p < 0.001). Similarly, *S. mansoni* infection showed an increased risk of bladder morbidity (cOR = 1.5; 95% CI: [1.1–2.0]; p = 0.006). However, after adjustment, there was a strong interaction between both *Schistosoma* infections with bladder morbidity (Interaction OR=2.4; 95% CI: [1.3–4.4]; p = 0.006), and the individual infections were no longer significant in the adjusted model (*S. haematobium*: aOR = 1.2 (95% CI: [0.8–1.9]; p = 0.451); *S. mansoni*: aOR = 0.8 (95% CI: [0.5–1.3]; p = 0.37) (S1 Table). Therefore, we performed a stratified analysis by infection status which confirmed that co-infected participants (*S. mansoni* + *S. haematobium*) had a significantly higher risk of bladder morbidity (cOR = 2.2; 95% CI: [1.5–3.2]; p < 0.001) compared to those with single infections. Furthermore, after adjusting for age and & gender, the risk of bladder morbidity remained significantly higher among participants with mixed infection (aOR =2.3, 95% CI: 1.5–3.5; p < 0.001) compared to those with single infections.

For liver morbidity, neither *S. haematobium* (OR = 1.5; 95% CI: [1.1-2.0]; p = 0.006) nor *S. mansoni* (OR = 1.5; 95% CI: [1.1-2.0]; p = 0.006) infection increased the risk of bladder morbidity (OR = 1.5; 95% CI: [1.1-2.0]; p = 0.006) in the unadjusted analysis. After adjustment, there was no significant interaction between liver morbidity and both infections (OR=1.4 [95% CI: 0.9-2.0], p = 0.125). Gender was also not a predictor of liver morbidity (OR=0.9 [0.7-1.2], p = 0.553). Therefore, we adjusted for age only, which showed no significant association between liver morbidity and *S. mansoni* (OR=1.3 [95% CI: 1.0-1.8], p = 0.062) or *S. haematobium* (OR=1.0 [95% CI: 0.7-1.4], p = 0.975).

## Discussion

The current study used ultrasound to assess schistosomiasis-related morbidity in a setting where *S. haematobium* and *S. mansoni* are co-endemic, thus contributing to a better understanding of the effect of mixed infection on bladder and liver morbidity patterns.

Overall, we found a high prevalence of both bladder (68%) and liver (64.6%) schistosomiasis-specific morbidity, which indicates a hyperendemicity profile for the disease in this study area, given their high prevalence. Previous studies conducted in mono or co-endemic conditions have shown that the prevalence of morbidity related to schistosomiasis can vary considerably across different regions [20,22,27,33,57,31]. The difference in prevalence may be attributed to variations in study populations and local factors such as the extent of schistosomiasis transmission or a history of mass treatment [19,20]. Our study was conducted in a praziquantel-naïve population exposed to infected water daily through farming and other at-risk water contact activities, which could explain the high prevalence of schistosomiasis-related morbidity.

The prevalence of bladder morbidity was found to be higher in participants with mixed infections compared to those with a single *S. haematobium* infection. In contrast, no difference in the prevalence rate of liver morbidity was observed between those coinfected compared to those with a single *S. mansoni* infection. This agrees with a similar study from Mali [35] in which the authors reported higher bladder morbidity in mixed compared to single *S. haematobium* infections, even if lower liver morbidity was found in mixed compared to single *S. mansoni* infections. Conversely to our findings, a lower *S. haematobium*-associated urogenital morbidity was shown in the coinfected participants in Kenya relative to single *S. haematobium* infections [54].

Additionally, while other similar studies in co-endemic areas have found that the prevalence of morbidity linked to *S. haematobium* appears to increase with co-infection, and at the same time, that related to *S. mansoni* tends to decrease or conversely [21,23,35], our study showed a similar trend in the prevalence of bladder and liver morbidity. This interesting result requires further investigation.

Interestingly, nearly a fourth of individuals (21.6%) who presented with morbidity were negative for the infection. This result must be interpreted in the context of the limitations of parasitological techniques in detecting low infection or other underlying disease conditions which have not been investigated in this study [58–60]. Therefore, it is possible that some of those who tested negative were actually infected, which has implications for recommendations regarding control measures. However, given the profile of the research area, these results are not surprising. The observed morbidity in this group may be due to the long-term, chronic effects of past infection rather than active infection.

Demographic (age and sex) patterns of schistosomiasis-associated morbidity in our study contrasted with those from other studies [22–26]. The likelihood of both bladder and liver morbidity increases with age. In fact, we found that age was correlated with the risk of both bladder and liver morbidity. For instance, in contrast to other studies where children were more at risk of bladder morbidity while adults were more at risk for hepatic fibrosis [22–25], our study found that adults were systematically more at higher risk of both bladder and liver morbidity. Regarding sex, no significant difference was found between males and females, which differ from other studies that reported males being more at risk for bladder morbidity and hepatic fibrosis than females [22–26].

Our findings confirmed that mixed infection increased significantly the risk of bladder morbidity (aOR=2.3; p < 0.001). Our results on bladder morbidity are in line with a study conducted in Mali [35], which found that bladder pathology was increased in co-infections over *S. haematobium* mono-infections. However, there is a contrast between one study in Kenya [54] and another in Senegal [22], which found that bladder pathology decreased in co-infections. This suggests a protective effect of current *S. mansoni* infection on bladder morbidity. Increased bladder morbidity could be explained by interspecies competition. It has been hypothesized that males of *S. haematobium* outcompete males *of S. mansoni*, leading to a shift in worm pair location. This results in preferential egg entrapment and pathology in the bladder rather than the liver [34,61,61–65]. In the study, as mentioned earlier conducted in Senegal [22], the reduced bladder morbidity was explained by the production of more ectopic *S. mansoni* than ectopic *S. haematobium* eggs resulting from heterologous pairs made of the competitively stronger male of *S. haematobium* and *S. mansoni* female. It was speculated that these heterologous pairs would produce less pathogenic eggs than homologous *S. haematobium* worm pairs or that their eggs would deviate to other sites [20,22,34,66]. Of note, nothing is known yet about the pathogenicity of eggs from heterologous pairs, and further studies are warranted to understand this phenomenon better. Another hypothesis from the Senegalese study is the chronology of the invasion by each *Schistosoma* species [67–69]. In Senegal, *S. haematobium* transmission occurred later than that of *S. mansoni*, raising the possibility that cross-resistance from *S. mansoni* may have conferred some protection in individuals with mixed infections. This hypothesis could not be evaluated in our study, as ectopic eggs were not examined and the historical sequence of *Schistosoma* species introduction in the study area is unknown. Unlike the protective effect reported in Senegal [23], we observed that co-infection with *S. haematobium* and *S. mansoni* was associated with an increased risk of bladder morbidity. The mechanisms underlying this association remain unclear, highlighting the need for further studies exploring immune-pathological interactions*.*

Regarding liver morbidity, our results showed no significant risk factor except age, which is in line with the aforementioned study in Senegal [22] but in contrast with the aforementioned study in Mali [35] and another study in Cameroun [34] where a decreased risk for abnormal liver imaging and hepatomegaly was found for coinfected versus *S. mansoni* mono-infected. More research is needed to better understand the effect of mixed infection on liver morbidity.

We acknowledge that our study has certain limitations. Firstly, the present study included the entire population, including children less than two years of age. As the anatomical structures of the liver and bladder are still developing at this age, it is debatable whether the observed prevalence of lesions in this group is accurate. However, given the high endemicity of schistosomiasis in our research area, with infections occurring at a time when organs are still developing, the potential severity of tissue lesions could be significantly greater. Furthermore, we did not consider other infections that can cause hepatomegaly and splenomegaly, particularly in children, such as malaria [13], which is also prevalent in the study area. Nevertheless, given that only 25 children under two years old were included in our study (13 of whom showed lesions), we believe this would not influence the overall age trend of bladder and liver morbidity. Another limitation was that the parasitological techniques used (the Kato-Katz method and filtration technique for analysis of the stool and urine samples, respectively) were ineffective in detecting low infection or after treatment with praziquantel. This likely resulted in an underestimation of the prevalence. Therefore, other techniques are necessary. In this study, however, we improved the sensitivity of these methods by collecting multiple samples: two stool samples and two urine samples on consecutive days.

In summary, the results of this study could have implications for disease control. Currently, mass drug administration with praziquantel tackles disease morbidity in school-aged children. However, our results showed that this morbidity is common in all ages and increases with age, suggesting a need for control measures also for adults. This includes regular MDA with praziquantel, considering the adult population. Community-based interventions like improved WASH infrastructure and health education to encourage behavioural change are also crucial for long-term control.

## Supporting information

S1 Data Morbidity database. (XLSX)

S1 Table The interaction model evaluated the effect of mixed infection compared to *S. haematobium* and *S. mansoni* mono-infection on bladder morbidity and liver morbidity. (XLSX)
